# (3*S*,N*R*)-3-Hydroxy­methyl-2-methyl-2-(3-methyl­but-2-en-1-yl)-1,2,3,4-tetra­hydro­isoquinolinium bromide–1,1′-bi-2-naphthol (1/1)

**DOI:** 10.1107/S1600536810007944

**Published:** 2010-03-06

**Authors:** Xiao-Qing Wu, Qing-Chun Huang, Xiao-Zhen Chen, Guo-You Li, Guo-Lin Zhang

**Affiliations:** a Chengdu Institute of Biology, Chinese Academy of Sciences, Chengdu 610041, People’s Republic of China; b Chengdu Institute of Organic Chemistry, Chinese Academy of Sciences, Chengdu 610041, People’s Republic of China

## Abstract

In the title compound, C_16_H_24_NO^+^·Br^−^·C_20_H_14_O_2_, the *N*-hetero­cyclic six-membered ring assumes a half-chair conformation. The two naphthalene ring systems are nearly perpendicular to one another, making a dihedral angle of 89.5 (2)°. Inter­molecular O—H⋯Br hydrogen bonding helps to stabilize the crystal structure.

## Related literature

For the optical properties of binaphthalen-2-ol, see: Tayama & Tanaka (2007 ▶). For related structures with nearly perpendicular naphthyl rings, see: Fukushima *et al.* (1999 ▶); Mori *et al.* (1993 ▶). For the synthesis, see: Schultz *et al.* (1998 ▶); Tayama & Tanaka (2007 ▶).
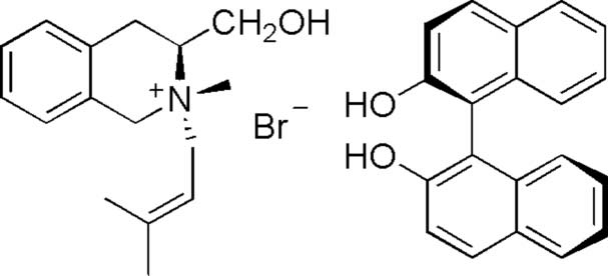


## Experimental

### 

#### Crystal data

C_16_H_24_NO^+^·C_20_H_14_O_2_·Br^−^·C_20_H_14_O_2_
*M* *_r_* = 612.58
Monoclinic, 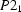

*a* = 11.3972 (5) Å
*b* = 10.1329 (4) Å
*c* = 13.8151 (5) Å
β = 107.976 (1)°
*V* = 1517.58 (11) Å^3^
*Z* = 2
Mo *K*α radiation
μ = 1.39 mm^−1^
*T* = 153 K
0.42 × 0.31 × 0.18 mm

#### Data collection

Rigaku R-AXIS RAPID diffractometer
Absorption correction: multi-scan (*ABSCOR*; Higashi, 1995 ▶) *T*_min_ = 0.593, *T*_max_ = 0.788
15042 measured reflections
6319 independent reflections
4162 reflections with *I* > 2σ(*I*)
*R*_int_ = 0.039

#### Refinement

*R*[*F* ^2^ > 2σ(*F* ^2^)] = 0.045
*wR*(*F* ^2^) = 0.180
*S* = 1.00
6319 reflections
386 parameters
1 restraint
H atoms treated by a mixture of independent and constrained refinement
Δρ_max_ = 0.68 e Å^−3^
Δρ_min_ = −0.68 e Å^−3^
Absolute structure: Flack (1983 ▶), 2637 Friedel pairs
Flack parameter: −0.002 (13)

### 

Data collection: *PROCESS-AUTO* (Rigaku, 1998 ▶); cell refinement: *PROCESS-AUTO*; data reduction: *CrystalStructure* (Rigaku/MSC, 2002 ▶); program(s) used to solve structure: *SHELXS97* (Sheldrick, 2008 ▶); program(s) used to refine structure: *SHELXL97* (Sheldrick, 2008 ▶); molecular graphics: *ORTEP-3 for Windows* (Farrugia, 1997 ▶); software used to prepare material for publication: *WinGX* (Farrugia, 1999 ▶).

## Supplementary Material

Crystal structure: contains datablocks global, I. DOI: 10.1107/S1600536810007944/xu2720sup1.cif

Structure factors: contains datablocks I. DOI: 10.1107/S1600536810007944/xu2720Isup2.hkl

Additional supplementary materials:  crystallographic information; 3D view; checkCIF report

## Figures and Tables

**Table 1:** Hydrogen-bond geometry (Å, °)

*D*—H⋯*A*	*D*—H	H⋯*A*	*D*⋯*A*	*D*—H⋯*A*
O1—H1*O*⋯Br1^i^	0.74 (7)	2.56 (7)	3.294 (5)	170 (6)
O2—H2*O*⋯Br1	0.83 (6)	2.50 (6)	3.291 (4)	161 (6)
O3—H3*O*⋯Br1^ii^	0.85 (10)	2.50 (10)	3.344 (4)	174 (9)

Symmetry codes: (i) 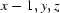; (ii) 
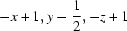
.

**Fig. 1.**
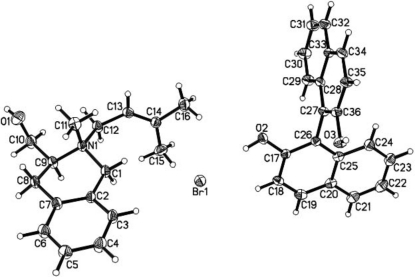
View of the molecule of (1) showing the atom-numbering scheme. Displacement ellipsoids are drawn at the 30% probability level.

## supplementary crystallographic information

### Comment

The molecular is formed with (3*S*,*NR*)-1,2,3,4- tetrahydro-3-(hydroxymethyl)-2-methyl-2-(3-methylbut-2-en-1-yl) isoquinolinium bromide and (*S*)-1,1'-binaphthalenol. The molecular structure is shown in Fig. 1.

In the molecular, the N-Chiral molecue contain two six-membered rings A (atom C2–C7) and B (C1,C2,C7–C9,N1), and the ring A is benzene ring, the ring B exists in half-chair conformation with N1, C9 at the flap. The C1–C8; C8,C9,N1 and C1,N1,C9 respectively form least square plane D, E, F, and the dihedral angel between the plane D and E is -47.4 (7)°[C7—C8—C9—N1], between the plane E and F is 63.9 (6)° [C1—N1—C9—C8], between the plane D and F is -51.6 (6)° [C9—N1—C1—C2]. The N-Chiral QASs molecule have two chiral center. The optical resolution can be efficiently carried out by an isomerization/crystallization method utilizing the axial chiral diol (*S*)-1,1'-binaphthyl-2,2'-diol (Tayama & Tanaka, 2007). The two naphthyl rings of (*S*)-1,1'-binaphthyl-2,2'-diol are approximately perpendicular to each other (Fig. 1) as observed in crystals (Mori *et al.*, 1993; Fukushima *et al.*, 1999). Intermolacular O—H···Br hydrogen bonding present in the crystal structure (Table 1).

### Experimental

(3*S*)-(2-Methyl-1,2,3,4-tetrahydroisoquinolin-3-yl)methanol (Schultz *et al.*, 1998) (1 mmol) and 3-methylbut-2-en-1-yl bromide (2 mmol) were dissolved in absolute acetonitrile (5 ml), refluxed for 42 h. After being cooled to room temperature, the excess 3-methylbut-2-en-1-yl bromide and acetonitrile were removed under reduced pressure. The residue was purified by flash chromatography eluted with ethyl acetate/methanol (8:1) to afford the diastereomeric mixtures of the salt. The two diastereomers of the salt was separated by Tayama and Tanaka's Method (Tayama & Tanaka, 2007).

### Refinement

Hydroxy H atoms were located in a difference Fourier map and were refined isotropically. Other H atoms were placed in calculated positions with C—H = 0.95-1.00 Å, and refined in riding mode with U_iso_(H) = 1.2U_eq_(C).

### Crystal data

**Table:**

C_16_H_24_NO^+^·C_20_H_14_O_2_·Br^−^·C_20_H_14_O_2_	*F*(000) = 640
*M**_r_* = 612.58	*D*_x_ = 1.341 Mg m^−^^3^
Monoclinic, *P*2_1_	Mo *K*α radiation, λ = 0.71073 Å
Hall symbol: P 2yb	Cell parameters from 10834 reflections
*a* = 11.3972 (5) Å	θ = 3.1–27.5°
*b* = 10.1329 (4) Å	µ = 1.39 mm^−^^1^
*c* = 13.8151 (5) Å	*T* = 153 K
β = 107.976 (1)°	Block, colorless
*V* = 1517.58 (11) Å^3^	0.42 × 0.31 × 0.18 mm
*Z* = 2	

### Data collection

**Table:**

Rigaku R-AXIS RAPID diffractometer	6319 independent reflections
Radiation source: Rotating Anode	4162 reflections with *I* > 2σ(*I*)
graphite	*R*_int_ = 0.039
ω scans	θ_max_ = 27.5°, θ_min_ = 3.1°
Absorption correction: multi-scan (*ABSCOR*; Higashi, 1995)	*h* = −14→14
*T*_min_ = 0.593, *T*_max_ = 0.788	*k* = −12→13
15042 measured reflections	*l* = −17→17

### Refinement

**Table:**

Refinement on *F*^2^	Hydrogen site location: inferred from neighbouring sites
Least-squares matrix: full	H atoms treated by a mixture of independent and constrained refinement
*R*[*F*^2^ > 2σ(*F*^2^)] = 0.045	*w* = 1/[σ^2^(*F*_o_^2^) + (0.1*P*)^2^ + 1.096*P*] where *P* = (*F*_o_^2^ + 2*F*_c_^2^)/3
*wR*(*F*^2^) = 0.180	(Δ/σ)_max_ < 0.001
*S* = 1.00	Δρ_max_ = 0.68 e Å^−^^3^
6319 reflections	Δρ_min_ = −0.68 e Å^−^^3^
386 parameters	Extinction correction: *SHELXL97* (Sheldrick, 2008), Fc^*^=kFc[1+0.001xFc^2^λ^3^/sin(2θ)]^-1/4^
1 restraint	Extinction coefficient: 0.031 (3)
Primary atom site location: structure-invariant direct methods	Absolute structure: Flack (1983), 2637 Friedel pairs
Secondary atom site location: difference Fourier map	Flack parameter: −0.002 (13)

### Special details

**Table:**

Experimental. IR (KBr): 3421, 2976, 2928, 1638, 1454, 1082, 766 cm^-1^; ^13^C-NMR (150 MHz, CDCl_3_): 149.2; 130.0; 128.9; 128.7; 127.5; 127.3; 126.1; 110.8; 66.8; 61.8; 59.4; 44.1;27.6; 26.6; 19.6. HR-ESI-MS: 246.1850 ([M–Br]^+^, C_16_H_24_NO^+^; calc. 246.1852).
Geometry. All esds (except the esd in the dihedral angle between two l.s. planes) are estimated using the full covariance matrix. The cell esds are taken into account individually in the estimation of esds in distances, angles and torsion angles; correlations between esds in cell parameters are only used when they are defined by crystal symmetry. An approximate (isotropic) treatment of cell esds is used for estimating esds involving l.s. planes.
Refinement. Refinement of *F*^2^ against ALL reflections. The weighted *R*-factor wR and goodness of fit *S* are based on *F*^2^, conventional *R*-factors *R* are based on *F*, with *F* set to zero for negative *F*^2^. The threshold expression of *F*^2^ > σ(*F*^2^) is used only for calculating *R*-factors(gt) etc. and is not relevant to the choice of reflections for refinement. *R*-factors based on *F*^2^ are statistically about twice as large as those based on *F*, and *R*- factors based on ALL data will be even larger.

### Fractional atomic coordinates and isotropic or equivalent isotropic displacement parameters (Å^2^)

**Table:**

	*x*	*y*	*z*	*U*_iso_*/*U*_eq_	
Br1	0.24874 (5)	0.38085 (9)	0.48816 (4)	0.0613 (2)	
O1	−0.4886 (4)	0.3748 (8)	0.4353 (4)	0.0836 (17)	
O2	0.4637 (4)	0.2940 (4)	0.6986 (3)	0.0518 (10)	
O3	0.7346 (4)	0.1223 (4)	0.6721 (3)	0.0472 (9)	
N1	−0.2234 (4)	0.4165 (4)	0.4580 (3)	0.0380 (10)	
C1	−0.1158 (5)	0.4553 (7)	0.4230 (4)	0.0459 (13)	
H1A	−0.0485	0.3905	0.4496	0.055*	
H1B	−0.0850	0.5425	0.4522	0.055*	
C2	−0.1468 (5)	0.4623 (6)	0.3087 (4)	0.0424 (12)	
C3	−0.0493 (5)	0.4536 (7)	0.2679 (4)	0.0571 (17)	
H3	0.0319	0.4344	0.3104	0.068*	
C4	−0.0728 (6)	0.4734 (8)	0.1637 (5)	0.0650 (18)	
H4	−0.0072	0.4692	0.1350	0.078*	
C5	−0.1902 (6)	0.4989 (8)	0.1029 (4)	0.0628 (18)	
H5	−0.2053	0.5144	0.0322	0.075*	
C6	−0.2862 (6)	0.5022 (7)	0.1429 (4)	0.0539 (15)	
H6	−0.3676	0.5175	0.0994	0.065*	
C7	−0.2658 (5)	0.4833 (6)	0.2466 (4)	0.0438 (12)	
C8	−0.3700 (5)	0.4857 (6)	0.2910 (4)	0.0449 (12)	
H8A	−0.4153	0.4011	0.2751	0.054*	
H8B	−0.4278	0.5570	0.2577	0.054*	
C9	−0.3304 (5)	0.5070 (6)	0.4055 (4)	0.0437 (12)	
H9	−0.2974	0.5990	0.4173	0.052*	
C10	−0.4372 (6)	0.5020 (8)	0.4468 (5)	0.0599 (17)	
H10A	−0.4092	0.5261	0.5198	0.072*	
H10B	−0.5007	0.5665	0.4103	0.072*	
C11	−0.2518 (6)	0.2732 (6)	0.4345 (5)	0.0513 (14)	
H11A	−0.2676	0.2580	0.3615	0.062*	
H11B	−0.3249	0.2485	0.4535	0.062*	
H11C	−0.1814	0.2195	0.4732	0.062*	
C12	−0.1876 (5)	0.4348 (7)	0.5745 (4)	0.0473 (13)	
H12A	−0.1747	0.5300	0.5903	0.057*	
H12B	−0.2573	0.4053	0.5976	0.057*	
C13	−0.0749 (5)	0.3619 (7)	0.6328 (3)	0.0468 (12)	
H13	−0.0843	0.2699	0.6414	0.056*	
C14	0.0378 (5)	0.4109 (6)	0.6745 (4)	0.0476 (14)	
C15	0.0735 (7)	0.5505 (7)	0.6669 (5)	0.0665 (18)	
H15A	0.1320	0.5553	0.6278	0.080*	
H15B	0.1122	0.5860	0.7353	0.080*	
H15C	−0.0001	0.6024	0.6326	0.080*	
C16	0.1391 (6)	0.3246 (8)	0.7354 (5)	0.0677 (19)	
H16A	0.1079	0.2346	0.7362	0.081*	
H16B	0.1700	0.3583	0.8052	0.081*	
H16C	0.2062	0.3240	0.7049	0.081*	
C17	0.5660 (4)	0.3709 (6)	0.7316 (3)	0.0363 (9)	
C18	0.5603 (5)	0.5051 (5)	0.7061 (4)	0.0399 (11)	
H18	0.4852	0.5432	0.6653	0.048*	
C19	0.6647 (6)	0.5809 (5)	0.7408 (4)	0.0467 (13)	
H19	0.6610	0.6715	0.7223	0.056*	
C20	0.7767 (5)	0.5286 (5)	0.8031 (4)	0.0412 (12)	
C21	0.8858 (6)	0.6058 (6)	0.8398 (5)	0.0541 (14)	
H21	0.8838	0.6967	0.8223	0.065*	
C22	0.9932 (6)	0.5514 (7)	0.8997 (5)	0.0615 (17)	
H22	1.0651	0.6043	0.9240	0.074*	
C23	0.9971 (5)	0.4173 (6)	0.9254 (4)	0.0531 (16)	
H23	1.0720	0.3799	0.9671	0.064*	
C24	0.8950 (5)	0.3401 (6)	0.8912 (4)	0.0463 (13)	
H24	0.8998	0.2493	0.9093	0.056*	
C25	0.7809 (4)	0.3932 (5)	0.8287 (3)	0.0356 (9)	
C26	0.6748 (4)	0.3142 (5)	0.7930 (3)	0.0327 (10)	
C27	0.6773 (4)	0.1713 (5)	0.8183 (3)	0.0341 (10)	
C28	0.6523 (4)	0.1276 (5)	0.9080 (3)	0.0339 (10)	
C29	0.6179 (5)	0.2139 (6)	0.9743 (4)	0.0460 (13)	
H29	0.6121	0.3057	0.9597	0.055*	
C30	0.5926 (6)	0.1699 (7)	1.0584 (4)	0.0548 (15)	
H30	0.5694	0.2309	1.1015	0.066*	
C31	0.6007 (6)	0.0339 (7)	1.0825 (4)	0.0555 (15)	
H31	0.5831	0.0035	1.1416	0.067*	
C32	0.6337 (5)	−0.0525 (6)	1.0211 (4)	0.0478 (13)	
H32	0.6395	−0.1438	1.0376	0.057*	
C33	0.6598 (5)	−0.0093 (5)	0.9326 (4)	0.0369 (10)	
C34	0.6936 (5)	−0.0977 (5)	0.8673 (4)	0.0413 (11)	
H34	0.6988	−0.1891	0.8831	0.050*	
C35	0.7191 (5)	−0.0567 (5)	0.7825 (4)	0.0404 (11)	
H35	0.7426	−0.1185	0.7402	0.048*	
C36	0.7100 (5)	0.0801 (5)	0.7579 (4)	0.0364 (10)	
H1O	−0.542 (6)	0.378 (9)	0.455 (5)	0.061 (18)*	
H2O	0.405 (6)	0.329 (6)	0.655 (5)	0.053 (18)*	
H3O	0.741 (9)	0.057 (10)	0.635 (7)	0.12 (4)*	

### Atomic displacement parameters (Å^2^)

**Table:**

	*U*^11^	*U*^22^	*U*^33^	*U*^12^	*U*^13^	*U*^23^
Br1	0.0427 (3)	0.0882 (5)	0.0533 (3)	0.0066 (3)	0.0154 (2)	0.0232 (4)
O1	0.051 (3)	0.115 (5)	0.102 (4)	0.008 (4)	0.049 (3)	0.028 (4)
O2	0.036 (2)	0.049 (2)	0.059 (2)	−0.0024 (17)	−0.0014 (18)	0.014 (2)
O3	0.059 (3)	0.044 (2)	0.045 (2)	−0.0069 (18)	0.0260 (18)	−0.0015 (18)
N1	0.031 (2)	0.048 (3)	0.0339 (18)	0.0060 (17)	0.0091 (15)	0.0000 (18)
C1	0.032 (3)	0.070 (4)	0.035 (2)	0.004 (2)	0.009 (2)	0.002 (2)
C2	0.030 (3)	0.064 (4)	0.032 (2)	−0.001 (2)	0.0084 (19)	0.001 (2)
C3	0.033 (3)	0.099 (5)	0.041 (3)	0.010 (3)	0.012 (2)	0.006 (3)
C4	0.047 (4)	0.104 (6)	0.050 (3)	−0.001 (4)	0.023 (3)	−0.011 (4)
C5	0.060 (4)	0.099 (5)	0.031 (3)	−0.005 (4)	0.017 (3)	−0.007 (3)
C6	0.051 (4)	0.074 (4)	0.033 (3)	−0.001 (3)	0.008 (2)	−0.008 (3)
C7	0.038 (3)	0.054 (3)	0.036 (2)	0.003 (2)	0.008 (2)	−0.002 (2)
C8	0.031 (3)	0.063 (3)	0.039 (3)	0.009 (2)	0.007 (2)	0.002 (2)
C9	0.037 (3)	0.053 (3)	0.038 (3)	0.010 (2)	0.007 (2)	−0.001 (2)
C10	0.039 (3)	0.095 (5)	0.049 (3)	0.012 (3)	0.018 (2)	0.003 (3)
C11	0.053 (4)	0.046 (3)	0.052 (3)	0.003 (3)	0.013 (3)	−0.007 (3)
C12	0.042 (3)	0.068 (4)	0.031 (2)	0.005 (2)	0.010 (2)	0.001 (2)
C13	0.047 (3)	0.057 (3)	0.036 (2)	0.003 (3)	0.0123 (19)	0.006 (3)
C14	0.042 (3)	0.065 (4)	0.036 (2)	0.000 (2)	0.011 (2)	0.003 (3)
C15	0.069 (5)	0.065 (4)	0.064 (4)	−0.023 (3)	0.020 (3)	0.002 (3)
C16	0.047 (3)	0.095 (5)	0.055 (3)	0.002 (3)	0.007 (3)	0.009 (3)
C17	0.036 (2)	0.040 (3)	0.0346 (19)	−0.002 (2)	0.0119 (16)	0.003 (3)
C18	0.048 (3)	0.034 (3)	0.038 (2)	0.006 (2)	0.013 (2)	0.005 (2)
C19	0.063 (4)	0.034 (3)	0.046 (3)	0.000 (2)	0.021 (3)	−0.002 (2)
C20	0.053 (3)	0.035 (3)	0.040 (3)	−0.007 (2)	0.020 (2)	−0.007 (2)
C21	0.060 (4)	0.051 (3)	0.055 (3)	−0.017 (3)	0.023 (3)	−0.014 (3)
C22	0.054 (4)	0.069 (4)	0.062 (4)	−0.023 (3)	0.019 (3)	−0.018 (3)
C23	0.042 (3)	0.074 (5)	0.040 (2)	−0.008 (3)	0.008 (2)	−0.007 (3)
C24	0.043 (3)	0.057 (3)	0.038 (2)	−0.002 (2)	0.010 (2)	−0.002 (2)
C25	0.044 (2)	0.034 (2)	0.0299 (18)	−0.007 (2)	0.0132 (16)	−0.007 (2)
C26	0.034 (2)	0.035 (2)	0.028 (2)	−0.0021 (18)	0.0088 (17)	−0.0005 (18)
C27	0.039 (3)	0.033 (2)	0.030 (2)	0.0022 (19)	0.0096 (18)	0.0035 (19)
C28	0.036 (2)	0.032 (2)	0.033 (2)	0.0035 (19)	0.0102 (18)	0.004 (2)
C29	0.058 (3)	0.044 (3)	0.040 (3)	0.003 (2)	0.021 (2)	−0.001 (2)
C30	0.071 (4)	0.061 (4)	0.038 (3)	0.000 (3)	0.026 (3)	−0.002 (3)
C31	0.065 (4)	0.063 (4)	0.041 (3)	0.001 (3)	0.019 (3)	0.009 (3)
C32	0.053 (3)	0.051 (3)	0.038 (2)	−0.002 (3)	0.012 (2)	0.011 (2)
C33	0.034 (2)	0.041 (3)	0.035 (2)	0.000 (2)	0.0092 (19)	0.010 (2)
C34	0.043 (3)	0.034 (3)	0.043 (2)	0.003 (2)	0.0088 (19)	0.006 (2)
C35	0.050 (3)	0.030 (2)	0.043 (3)	0.001 (2)	0.018 (2)	0.001 (2)
C36	0.041 (3)	0.038 (3)	0.032 (2)	−0.002 (2)	0.0137 (19)	0.003 (2)

### Geometric parameters (Å, °)

**Table:**

O1—C10	1.404 (10)	C15—H15A	0.9800
O1—H1O	0.74 (7)	C15—H15B	0.9800
O2—C17	1.359 (6)	C15—H15C	0.9800
O2—H2O	0.83 (6)	C16—H16A	0.9800
O3—C36	1.367 (6)	C16—H16B	0.9800
O3—H3O	0.85 (10)	C16—H16C	0.9800
N1—C11	1.501 (7)	C17—C26	1.392 (6)
N1—C1	1.503 (7)	C17—C18	1.402 (8)
N1—C9	1.519 (6)	C18—C19	1.372 (8)
N1—C12	1.544 (6)	C18—H18	0.9500
C1—C2	1.510 (7)	C19—C20	1.405 (8)
C1—H1A	0.9900	C19—H19	0.9500
C1—H1B	0.9900	C20—C25	1.414 (8)
C2—C7	1.379 (7)	C20—C21	1.423 (8)
C2—C3	1.396 (8)	C21—C22	1.364 (10)
C3—C4	1.394 (8)	C21—H21	0.9500
C3—H3	0.9500	C22—C23	1.402 (9)
C4—C5	1.367 (9)	C22—H22	0.9500
C4—H4	0.9500	C23—C24	1.360 (8)
C5—C6	1.371 (8)	C23—H23	0.9500
C5—H5	0.9500	C24—C25	1.426 (7)
C6—C7	1.392 (7)	C24—H24	0.9500
C6—H6	0.9500	C25—C26	1.407 (7)
C7—C8	1.496 (8)	C26—C27	1.488 (7)
C8—C9	1.520 (7)	C27—C36	1.372 (7)
C8—H8A	0.9900	C27—C28	1.425 (6)
C8—H8B	0.9900	C28—C29	1.407 (7)
C9—C10	1.497 (8)	C28—C33	1.425 (7)
C9—H9	1.0000	C29—C30	1.356 (8)
C10—H10A	0.9900	C29—H29	0.9500
C10—H10B	0.9900	C30—C31	1.414 (9)
C11—H11A	0.9800	C30—H30	0.9500
C11—H11B	0.9800	C31—C32	1.352 (9)
C11—H11C	0.9800	C31—H31	0.9500
C12—C13	1.486 (7)	C32—C33	1.413 (7)
C12—H12A	0.9900	C32—H32	0.9500
C12—H12B	0.9900	C33—C34	1.407 (7)
C13—C14	1.331 (8)	C34—C35	1.355 (7)
C13—H13	0.9500	C34—H34	0.9500
C14—C16	1.485 (8)	C35—C36	1.424 (7)
C14—C15	1.485 (9)	C35—H35	0.9500
			
C10—O1—H1O	106 (7)	H15A—C15—H15B	109.5
C17—O2—H2O	114 (4)	C14—C15—H15C	109.5
C36—O3—H3O	111 (7)	H15A—C15—H15C	109.5
C11—N1—C1	109.0 (4)	H15B—C15—H15C	109.5
C11—N1—C9	113.1 (4)	C14—C16—H16A	109.5
C1—N1—C9	107.7 (4)	C14—C16—H16B	109.5
C11—N1—C12	108.1 (4)	H16A—C16—H16B	109.5
C1—N1—C12	108.9 (4)	C14—C16—H16C	109.5
C9—N1—C12	109.9 (4)	H16A—C16—H16C	109.5
N1—C1—C2	113.5 (4)	H16B—C16—H16C	109.5
N1—C1—H1A	108.9	O2—C17—C26	118.6 (5)
C2—C1—H1A	108.9	O2—C17—C18	120.3 (4)
N1—C1—H1B	108.9	C26—C17—C18	121.1 (5)
C2—C1—H1B	108.9	C19—C18—C17	119.2 (5)
H1A—C1—H1B	107.7	C19—C18—H18	120.4
C7—C2—C3	120.8 (5)	C17—C18—H18	120.4
C7—C2—C1	121.7 (5)	C18—C19—C20	121.9 (5)
C3—C2—C1	117.5 (5)	C18—C19—H19	119.0
C4—C3—C2	118.9 (5)	C20—C19—H19	119.0
C4—C3—H3	120.5	C19—C20—C25	118.2 (5)
C2—C3—H3	120.5	C19—C20—C21	122.7 (5)
C5—C4—C3	120.2 (6)	C25—C20—C21	119.1 (5)
C5—C4—H4	119.9	C22—C21—C20	121.0 (6)
C3—C4—H4	119.9	C22—C21—H21	119.5
C4—C5—C6	120.5 (5)	C20—C21—H21	119.5
C4—C5—H5	119.7	C21—C22—C23	119.9 (6)
C6—C5—H5	119.7	C21—C22—H22	120.1
C5—C6—C7	120.7 (6)	C23—C22—H22	120.1
C5—C6—H6	119.6	C24—C23—C22	120.9 (6)
C7—C6—H6	119.6	C24—C23—H23	119.6
C2—C7—C6	118.8 (5)	C22—C23—H23	119.6
C2—C7—C8	120.0 (5)	C23—C24—C25	121.0 (5)
C6—C7—C8	121.2 (5)	C23—C24—H24	119.5
C7—C8—C9	114.2 (4)	C25—C24—H24	119.5
C7—C8—H8A	108.7	C26—C25—C20	120.4 (4)
C9—C8—H8A	108.7	C26—C25—C24	121.5 (5)
C7—C8—H8B	108.7	C20—C25—C24	118.2 (5)
C9—C8—H8B	108.7	C17—C26—C25	119.1 (4)
H8A—C8—H8B	107.6	C17—C26—C27	119.6 (4)
C10—C9—N1	115.1 (5)	C25—C26—C27	121.3 (4)
C10—C9—C8	112.2 (5)	C36—C27—C28	119.0 (4)
N1—C9—C8	110.4 (4)	C36—C27—C26	120.1 (4)
C10—C9—H9	106.2	C28—C27—C26	120.8 (4)
N1—C9—H9	106.2	C29—C28—C33	117.3 (4)
C8—C9—H9	106.2	C29—C28—C27	123.0 (4)
O1—C10—C9	110.3 (5)	C33—C28—C27	119.8 (4)
O1—C10—H10A	109.6	C30—C29—C28	122.0 (5)
C9—C10—H10A	109.6	C30—C29—H29	119.0
O1—C10—H10B	109.6	C28—C29—H29	119.0
C9—C10—H10B	109.6	C29—C30—C31	120.4 (6)
H10A—C10—H10B	108.1	C29—C30—H30	119.8
N1—C11—H11A	109.5	C31—C30—H30	119.8
N1—C11—H11B	109.5	C32—C31—C30	119.6 (5)
H11A—C11—H11B	109.5	C32—C31—H31	120.2
N1—C11—H11C	109.5	C30—C31—H31	120.2
H11A—C11—H11C	109.5	C31—C32—C33	121.1 (6)
H11B—C11—H11C	109.5	C31—C32—H32	119.5
C13—C12—N1	114.3 (4)	C33—C32—H32	119.5
C13—C12—H12A	108.7	C34—C33—C32	122.0 (5)
N1—C12—H12A	108.7	C34—C33—C28	118.3 (4)
C13—C12—H12B	108.7	C32—C33—C28	119.7 (5)
N1—C12—H12B	108.7	C35—C34—C33	122.3 (5)
H12A—C12—H12B	107.6	C35—C34—H34	118.9
C14—C13—C12	127.4 (6)	C33—C34—H34	118.9
C14—C13—H13	116.3	C34—C35—C36	119.1 (5)
C12—C13—H13	116.3	C34—C35—H35	120.5
C13—C14—C16	120.2 (6)	C36—C35—H35	120.5
C13—C14—C15	125.0 (6)	O3—C36—C27	118.9 (4)
C16—C14—C15	114.8 (6)	O3—C36—C35	119.5 (4)
C14—C15—H15A	109.5	C27—C36—C35	121.6 (4)
C14—C15—H15B	109.5		
			
C11—N1—C1—C2	71.5 (6)	C22—C23—C24—C25	−0.3 (8)
C9—N1—C1—C2	−51.6 (6)	C19—C20—C25—C26	−0.4 (7)
C12—N1—C1—C2	−170.7 (5)	C21—C20—C25—C26	−179.6 (4)
N1—C1—C2—C7	24.1 (8)	C19—C20—C25—C24	179.5 (4)
N1—C1—C2—C3	−159.1 (5)	C21—C20—C25—C24	0.3 (7)
C7—C2—C3—C4	3.4 (10)	C23—C24—C25—C26	180.0 (5)
C1—C2—C3—C4	−173.4 (7)	C23—C24—C25—C20	0.1 (7)
C2—C3—C4—C5	−1.0 (12)	O2—C17—C26—C25	−179.1 (4)
C3—C4—C5—C6	−1.5 (12)	C18—C17—C26—C25	−0.8 (7)
C4—C5—C6—C7	1.8 (11)	O2—C17—C26—C27	1.7 (6)
C3—C2—C7—C6	−3.2 (10)	C18—C17—C26—C27	−179.9 (4)
C1—C2—C7—C6	173.5 (6)	C20—C25—C26—C17	0.3 (6)
C3—C2—C7—C8	177.0 (6)	C24—C25—C26—C17	−179.5 (4)
C1—C2—C7—C8	−6.3 (9)	C20—C25—C26—C27	179.5 (4)
C5—C6—C7—C2	0.6 (10)	C24—C25—C26—C27	−0.4 (7)
C5—C6—C7—C8	−179.6 (6)	C17—C26—C27—C36	91.4 (6)
C2—C7—C8—C9	18.2 (8)	C25—C26—C27—C36	−87.7 (6)
C6—C7—C8—C9	−161.6 (6)	C17—C26—C27—C28	−92.0 (6)
C11—N1—C9—C10	71.5 (6)	C25—C26—C27—C28	88.9 (6)
C1—N1—C9—C10	−167.9 (5)	C36—C27—C28—C29	−179.9 (5)
C12—N1—C9—C10	−49.4 (6)	C26—C27—C28—C29	3.5 (7)
C11—N1—C9—C8	−56.7 (6)	C36—C27—C28—C33	−1.0 (7)
C1—N1—C9—C8	63.9 (6)	C26—C27—C28—C33	−177.6 (4)
C12—N1—C9—C8	−177.6 (5)	C33—C28—C29—C30	0.0 (8)
C7—C8—C9—C10	−177.2 (5)	C27—C28—C29—C30	178.9 (5)
C7—C8—C9—N1	−47.4 (7)	C28—C29—C30—C31	0.2 (9)
N1—C9—C10—O1	−61.7 (6)	C29—C30—C31—C32	−0.1 (10)
C8—C9—C10—O1	65.6 (7)	C30—C31—C32—C33	−0.3 (10)
C11—N1—C12—C13	62.5 (6)	C31—C32—C33—C34	−179.6 (5)
C1—N1—C12—C13	−55.8 (6)	C31—C32—C33—C28	0.5 (8)
C9—N1—C12—C13	−173.5 (5)	C29—C28—C33—C34	179.8 (5)
N1—C12—C13—C14	102.3 (6)	C27—C28—C33—C34	0.8 (7)
C12—C13—C14—C16	176.7 (5)	C29—C28—C33—C32	−0.4 (7)
C12—C13—C14—C15	−2.3 (9)	C27—C28—C33—C32	−179.3 (5)
O2—C17—C18—C19	179.6 (5)	C32—C33—C34—C35	−179.8 (5)
C26—C17—C18—C19	1.2 (7)	C28—C33—C34—C35	0.1 (7)
C17—C18—C19—C20	−1.3 (8)	C33—C34—C35—C36	−0.7 (8)
C18—C19—C20—C25	0.9 (8)	C28—C27—C36—O3	−179.6 (4)
C18—C19—C20—C21	−180.0 (5)	C26—C27—C36—O3	−3.0 (7)
C19—C20—C21—C22	−179.7 (6)	C28—C27—C36—C35	0.3 (8)
C25—C20—C21—C22	−0.6 (8)	C26—C27—C36—C35	177.0 (5)
C20—C21—C22—C23	0.4 (9)	C34—C35—C36—O3	−179.5 (5)
C21—C22—C23—C24	0.1 (9)	C34—C35—C36—C27	0.6 (8)

### Hydrogen-bond geometry (Å, °)

**Table:**

*D*—H···*A*	*D*—H	H···*A*	*D*···*A*	*D*—H···*A*
O1—H1O···Br1^i^	0.74 (7)	2.56 (7)	3.294 (5)	170 (6)
O2—H2O···Br1	0.83 (6)	2.50 (6)	3.291 (4)	161 (6)
O3—H3O···Br1^ii^	0.85 (10)	2.50 (10)	3.344 (4)	174 (9)

Symmetry codes: (i) *x*−1, *y*, *z*; (ii) −*x*+1, *y*−1/2, −*z*+1.
